# Myositis Due to Parvovirus B19: A Case Report and Literature Review

**DOI:** 10.7759/cureus.83683

**Published:** 2025-05-07

**Authors:** Vivianne L Allsop, John Gaumnitz, Changhong Xing, Eric Goold, Julie Thomas

**Affiliations:** 1 Rheumatology, University of Utah Hospital, Salt Lake City, USA; 2 Internal Medicine, University of Utah Hospital, Salt Lake City, USA; 3 Pathology, University of Utah Hospital, Salt Lake City, USA

**Keywords:** b19 parvovirus infection, calf pain, elevated aldolase, myositis, viral myositis

## Abstract

Myositis is a condition characterized by muscle inflammation due to multiple etiologies, including autoimmune disease, medication, and infection. Viral myositis is most frequently seen in children after influenza infection, but Parvovirus B19 has also rarely been associated with myositis. We report the case of a 42-year-old male with a medical history of low back pain due to spondylosis who presented to the hospital with a one-month history of bilateral thigh and calf pain associated with subjective weakness and a one-week history of fevers. His wife and son were diagnosed with Parvovirus B19 infection one month before his symptoms began. The physical exam was notable for prominent tenderness to palpation of the anterior thigh muscles and calves and mild swelling around the ankles without intra-articular effusion. Strength was normal in all extremities. Lower extremity MRI revealed extensive multifocal, patchy, and feathery edema throughout the lower extremities. Aldolase, C-reactive protein (CRP), erythrocyte sedimentation rate (ESR), and Parvovirus B19 serum IgM were elevated, but creatine kinase, extended myositis antibody panel, and 3-hydroxy-3-methylglutaryl coenzyme A (HMG CoA) reductase antibody were normal. A vastus lateralis muscle biopsy demonstrated mild acute myopathic changes with patchy upregulation of major histocompatibility complex (MHC) class I antigens. Parvovirus B19 polymerase chain reaction (PCR) on the muscle tissue was positive. The patient was diagnosed with myositis due to Parvovirus B19 infection and started on scheduled naproxen. Five months later, his symptoms had completely resolved, and he no longer required naproxen. A literature review of PubMed, Ovid, Scopus, Web of Science, and Cochrane databases for English-language articles using the search terms “idiopathic inflammatory myopathy” OR “myositis” OR “inflammatory myopathy” OR “dermatomyositis” OR “polymyositis” AND “Parvovirus B19” OR “parvovirus” revealed six other cases of myositis associated with Parvovirus B19 infections, although two of the six cases had characteristics of dermatomyositis rather than viral myositis. Aside from the patient with dermatomyositis, one other patient had a muscle biopsy with positive Parvovirus B19 testing. To our knowledge, our case is the second description of biopsy-proven Parvovirus B19 myositis, and the first to describe specific histopathology. Further, ours is the first case of biopsy-proven Parvovirus B19 myositis to be successfully treated with non-steroidal anti-inflammatory drugs (NSAIDs) in an adult.

## Introduction

Viral infections can affect muscles as myalgias, myositis, or rhabdomyolysis and have been proposed as a trigger of idiopathic inflammatory myopathies. Viral myositis can be caused by various pathogens, including Influenza A and B, Enteroviruses (such as Coxsackieviruses A and B), Human T-lymphotropic virus type 1 (HTLV-1), and HIV [1]. The pathogenesis of viral myositis is not well understood. Direct viral invasion and immune-mediated damage to muscle are two proposed mechanisms. Clinical presentation and pathologic findings vary between viruses. Influenza B-associated myositis typically presents in children with severe bilateral calf pain [2], whereas Coxsackievirus B myositis classically presents in children as pleurodynia, resulting from chest wall muscle inflammation. Published biopsy findings vary from degenerative necrosis in Coxsackievirus B myositis to lymphocytic infiltration in HIV myositis. Parvovirus B19 has also been reported as a rare cause of viral myositis; however, the mechanism is not clearly understood [1].

Parvovirus B19 is a small, single-stranded, non-enveloped DNA virus from the Parvoviridae family, known to cause a wide range of clinical diseases in humans [3]. The virus is transmitted through respiratory secretions and then spreads via the bloodstream to the bone marrow, where it infects erythroid precursor cells, its primary target that permits replication [4]. Following replication, the virus is released from the marrow through cell lysis, resulting in high-level viremia and various clinical manifestations. 

Depending on the host’s immune and hematologic status, Parvovirus B19 infection can range from asymptomatic to a typical, self-limited, biphasic syndrome or even critical illness. The typical biphasic syndrome begins with a prodromal phase featuring fever, chills, headache, nausea, and myalgias, corresponding to viral replication and systemic spread. This is followed by a second, immune-mediated phase with largely age-specific symptoms. The second phase coincides with IgM and IgG formation, leading to viral clearance and formation of the immune complex, which can deposit in the skin and joints [5,6]. 

In healthy children, Parvovirus B19 typically causes erythema infectiosum, also known as *fifth disease*, which includes a prodromal illness followed by a distinctive *slapped cheek* rash and, occasionally, an erythematous maculopapular rash on the trunk and limbs [7,8]. In healthy adults, the initial prodrome leads to a symmetric polyarthritis of the small joints of the hands, feet, wrists, and ankles, mimicking another rheumatologic disease. This arthritis usually resolves within one to three weeks but can persist or recur for months to years [9,10]. Additionally, infections will often cause a low-level anemia in healthy hosts because of damage to erythropoiesis. However, in patients with chronic erythroid disorders, they can cause a severe, life-threatening anemia known as aplastic crisis [7]. 

While incompletely understood, it has been shown that Parvovirus B19 can directly infect non-erythroid cell types, causing inflammation and cell death [11]. Rare case reports have implicated the virus in various tissue-invasive conditions. Here, we report a muscle biopsy-proven case of Parvovirus B19-related myositis in an adult. To our knowledge, this is the first report of biopsy-proven Parvovirus B19-related myositis successfully treated with non-steroidal anti-inflammatory monotherapy, as well as the first published report to describe histopathologic findings on muscle biopsy in Parvovirus B19 myositis.

## Case presentation

**Figure 1:**
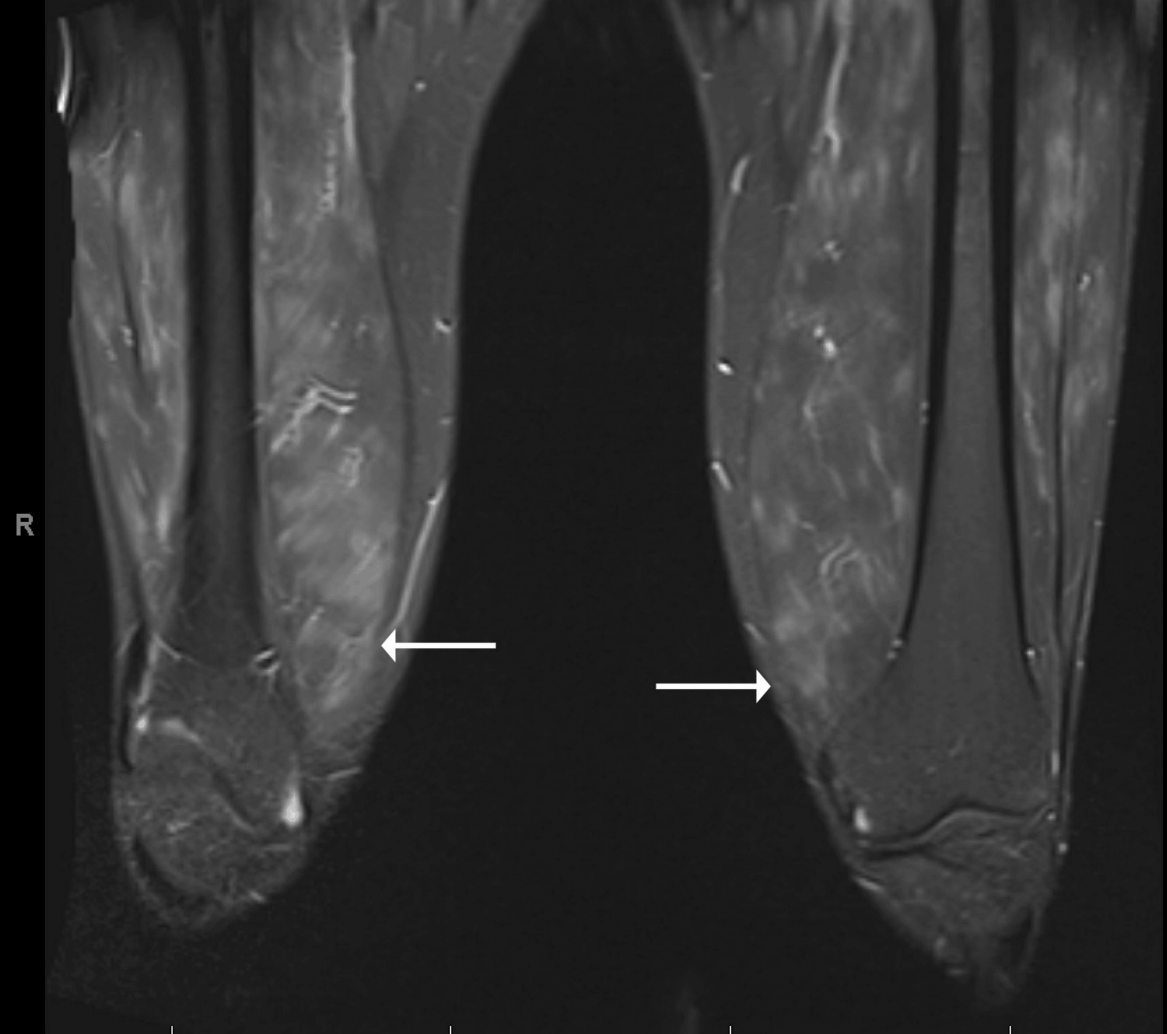
STIR sequence MRI of bilateral thighs shows diffuse feathery muscle edema and enhancement (arrows). STIR, short TI inversion recovery

A 42-year-old man with chronic low back pain due to spondylosis presented to the hospital with a one-month history of bilateral thigh and calf pain, accompanied by mild subjective weakness, and one week of fevers, measured at home at 103 °F. Two months prior, his wife and son were diagnosed with Parvovirus B19 infection. His son had erythema infectiosum, and his wife developed an inflammatory polyarthritis of the small joints that resolved with non-steroidal anti-inflammatory treatment. Before hospitalization, he visited an outpatient primary care clinic, then an emergency room, and was prescribed a steroid taper at each visit. Prednisone provided temporary relief with relapse of symptoms upon cessation. Initial vitals were normal, including temperature. Physical exam was notable for prominent tenderness to palpation of the anterior thigh and calf musculature bilaterally and mild swelling around the ankles without intra-articular effusion. Strength testing was normal in the neck and all extremities. MRI revealed extensive multifocal, patchy, and feathery edema throughout the proximal lower extremities (Figure 1). Laboratory findings included elevated aldolase, C-reactive protein (CRP), erythrocyte sedimentation rate (ESR), and Parvovirus B19 serum IgM, but normal creatine kinase and thyroid-stimulating hormone (TSH). Qualitative serum Parvovirus B19 PCR was positive; quantitative serum Parvovirus B19 PCR testing was not obtained. Tests for antinuclear and myositis-specific antibodies were negative, including anti-Smith ribonucleoprotein (anti-Smith/RNP), Sjögren syndrome antigen A 52 (SSA-52), Jo-1, polymyositis/scleroderma (PM/Scl), Mi-2, PL-7, PL-12, P155/140, EJ, Ku, signal recognition particle (SRP), OJ, Sjögren syndrome antigen A 60 (SSA-60), fibrillarin, SAE1, melanoma differentiation-associated protein 5 (MDA5), NXP2, TIF-1 gamma, and transcription intermediary factor 1 (TIF-1 gamma), and 3-hydroxy-3-methylglutaryl CoA (HMG CoA) reductase (Table 1). Chest X-ray was without abnormalities, including hilar adenopathy. Electromyography (EMG) could not be obtained during his inpatient stay. Routine cancer screening was encouraged, but extensive paraneoplastic evaluation was deferred due to his young age, clinical presentation, lack of malignancy in his family history, and high suspicion for a benign etiology. 

**Table 1:** Remarkable laboratory findings. WBC, white blood cell count; AST, aspartate aminotransferase; ALT, alanine aminotransferase; BUN, blood urea nitrogen; ESR, erythrocyte sedimentation rate; CRP, C-reactive protein; HIV 1,2 Combo Ag and Ab, human immunodeficiency virus 1 and 2 combination antigen and antibody test; ANA, antinuclear antibody; HMG CoA reductase IgG, 3-hydroxy-3-methylglutaryl coenzyme A reductase immunoglobulin G; TSH, thyroid stimulating hormone; ACE, angiotensin converting enzyme

Lab (blood/plasma/serum)	Patient’s value	Reference range
WBC	9.99	2.3-11.3 k/uL
Hemoglobin	12.6	14.8-17.8 g/dL
Platelets	297	159-439 k/uL
Lymphocyte #	1.1	1.30-3.60 k/uL
Creatinine	0.92	0.72-1.25 mg/dL
Calcium	8.9	8.4-10.5 mg/dL
AST	15	16-40 U/L
ALT	23	5-60 U/L
BUN	11	8-24 mg/dL
ESR	35	<15 mm/hr
CRP	9.1	0-0.8 mg/dL
Creatine Kinase	86	20-200 U/L
Aldolase	8.7	1.2-7.6 U/L
Parvovirus B19 IgM (serum)	6.33	<0.9 IV
Parvovirus B19 IgG (serum)	9.06	<0.9 IV
Qualitative Parvovirus B19 PCR (serum)	Positive	Negative
Quantitative Parvovirus B19 PCR (muscle)	2540 IU/mg	Not detected
HIV 1,2 Combo Ag and Ab	Negative	Negative
Hepatitis B total Core Ab	Negative	Negative
Hepatitis B surface Ag	Negative	Negative
Hepatitis C Ab Index by CIA	Negative	Negative
Respiratory viral panel	Negative	Negative
ANA	Negative	Negative
Myositis Antibody Profile	Negative	Negative
HMG CoA Reductase IgG	3	0-19 units
TSH	1.03	0.27-4.29 mU/L
ACE	17	16-85 U/L
25 OH Vitamin D 29 (30-90)	29	30-90 ng/mL

**Figure 2:**
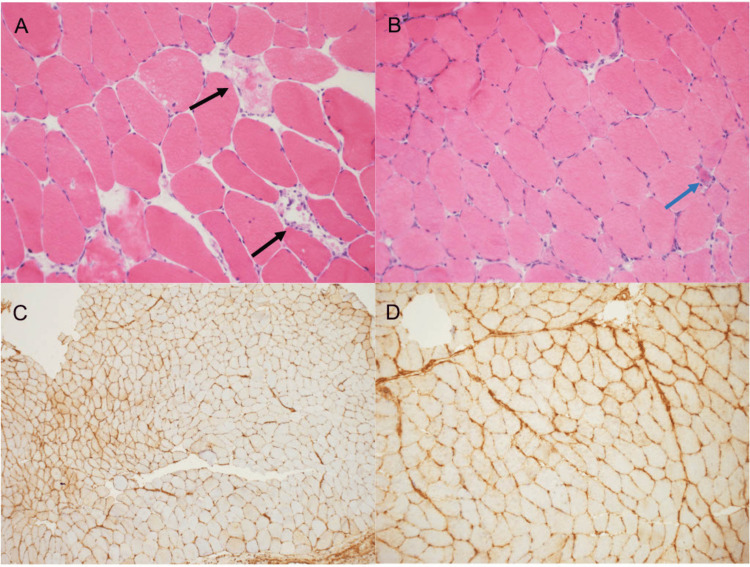
Histopathology of muscle biopsy. (A and B) H&E-stained cryostat sections showing mild fiber size variation, with occasional degenerating (black arrows) and regenerating (cyan arrows) fibers (A, 10x; B, 20x). (C and D) Immunohistochemical staining for MHC-I revealing patchy abnormal sarcoplasmic and sarcolemmal upregulation in a subset of fibers (C, 10x; D, 40x). MHC-I, major histocompatibility complex class I

Muscle biopsy was performed of the right vastus lateralis muscle and revealed mild acute myopathic changes showing occasional degenerating and regenerating myofibers (Figures 2A, 2B). There was no inflammation identified. However, immunohistochemistry demonstrated upregulation of MHC Class I antigens in a subset of myofibers, which may be suggestive of an inflammatory myopathy (Figures 2C, 2D). Quantitative Parvovirus B19 PCR was sent on the muscle biopsy sample and returned positive, and serum qualitative PCR confirmed high viral load. 

The patient was diagnosed with Parvovirus B19-associated myositis and treated with naproxen 250 mg three times daily (low dose selected due to patient preference), with improvement in his symptoms. Five months later, his symptoms were resolved, and he no longer required naproxen. 

## Discussion

Literature review methods

A search query was performed in the PubMed database on August 9, 2024, and in Ovid, Scopus, Web of Science, and Cochrane databases on March 23, 2025, for English-language articles using the search terms “idiopathic inflammatory myopathy” OR “myositis” OR “inflammatory myopathy” OR “dermatomyositis” OR “polymyositis” AND “Parvovirus B19” OR “parvovirus.” Inclusion criteria included: (1) description of a case of myositis with supporting evidence including at least one of the following: (i) abnormal MRI, (ii) abnormal EMG, (iii) abnormal muscle biopsy, (iv) abnormal creatine kinase or aldolase; (2) evidence of current or recent infection with Parvovirus B19 defined by serum Parvovirus B19 IgM positivity, serum Parvovirus nucleic acid amplification test (NAAT), or PCR positivity, or muscle biopsy with positive Parvovirus B19 PCR testing; (3) patient age of 18 or older. Exclusion criteria included: (1) not a full article, (2) full article not available in English, (3) plausible other cause of myositis reported. Dermatomyositis and polymyositis were included as search terms to explore the association more fully between inflammatory myositis and Parvovirus B19, although our patient did not have a presentation consistent with an idiopathic inflammatory myopathy. 

Pertinent data were extracted from each paper that met the inclusion and exclusion criteria, including patient age, sex, diagnostic studies, treatment, and clinical manifestations. Particular attention was paid to acute phase reactants (ESR and CRP), muscle enzymes, muscle biopsy, and Parvovirus B19 testing. Specific results are reported when available. Five articles reporting a total of six cases met the inclusion and exclusion criteria. These cases are summarized in Table 2 [12-16].

**Table 2:** Summary of Parvovirus B19 myositis cases identified in literature review. CRP, C-reactive protein; MRI, magnetic resonance imaging; ESR, erythrocyte sedimentation rate; PCR, polymerase chain reaction; IU, international units; IgM, immunoglobulin M; IgG, immunoglobulin G; EIU, enzyme immunoassay unit; PO, per os (by mouth)

Case	Author	Age	Sex	Disease manifestations	Diagnostics (Reference range or relationship to reference range in parentheses when reported)	Parvovirus B19 testing	Treatment	Follow-up course
1	Cakirca et al., 2013 [12]	38	M	Diffuse pain; fever	CRP 5.9 mg/dL (0-0.5); creatine kinase 51 mg/dL (39-308); MRI with muscle thickening and edema in bilateral gastrocnemius and semimembranosus muscles	Serum Parvovirus B19 IgM 76.9 U (positive)	Naproxen sodium 1100 mg per day	Reduced pain and normal inflammatory markers at day ten
2	Chevrel et al., 2000 [13]	48	F	Muscle pain and weakness; heliotrope rash	ESR 100; CRP normal; creatine kinase 724 IU/L (30-125); aldolase 22 IU/L (<7.6); muscle biopsy with perivascular lymphocytes and plasmocyte infiltration with necrosis	Parvovirus B19 PCR negative in serum x 4; Parvovirus B19 DNA detected in sample from muscle biopsy	Prednisone 20 mg per day and Methotrexate	Remained symptomatic four months after diagnosis. Tapered off medication after two years and remained in remission for at least three years
3	Ichinose et al., 2004 [14]	36	M	Severe myalgia and weakness of the bilateral lower extremities	CRP 0.98 mg/dL (normal); Creatine kinase 70 U/l (normal); aldolase 3.6 U/l (normal); MRI with increased signal in muscles and fascia of both soleus and gastrocnemius muscles	Serum Parvovirus B19 IgM 3.3 EIU/mL (<0.8)	Loxoprofen Sodium 180 mg three times per day	Parvovirus IgM level declined within two months
4	Magro et al., 2000 [15]	21	F	Classical Gottrón's papules; symmetrical proximal muscle weakness	Elevated creatine kinase	Serum Parvovirus B19 IgM positive	Not reported	Lost to follow-up
5	Oliver et al., 2012 [16]	42	M	Calf and thigh pain with weakness; ankle swelling, forearm pain	CRP elevated; creatine kinase normal; MRI with soleus muscle edema; muscle biopsy histology non-diagnostic	Parvovirus B19 PCR 32,900 cop/m from muscle biopsy	Prednisolone	Significant clinical response within two weeks
6	Oliver et al., 2012 [16]	46	M	Bilateral elbow pain; pain in both calves	ESR 119 mm/hour (Elevated); CRP 34 mg/L; creatine kinase 187 IU/L; MRI with intramuscular edema in both legs	Serum Parvovirus B19 IgM positive; serum Parvovirus PCR 66,400 cop/m	Prednisolone 40 mg PO daily	Significantly improved in one week

Case characteristics

Cases 2 and 4 [13,15] had characteristics of dermatomyositis rather than viral myositis, including classic dermatologic findings of heliotrope rash and Gottron's papules, respectively. Case 2 [13] also had perivascular lymphocytic infiltrate on muscle biopsy, a typical finding in dermatomyositis. Cases 1, 3, 5, and 6 [12,14,16] describe viral myositis from Parvovirus B19. Three of the four cases describe muscle pain as a prominent symptom, particularly in the lower extremities. Each of these four cases had muscle edema on MRI. None reported significantly elevated creatine kinase, but three had elevated inflammatory markers. Only case 5 [16] reported a muscle biopsy with non-diagnostic histology but positive Parvovirus B19 PCR sent on muscle tissue. Cases 1 and 3 [12,14] were managed with NSAID monotherapy, and cases 5 and 6 [16] were treated with glucocorticoids.

Parvovirus B19 as a cause of viral myositis

Including our patient, five of the seven patients in this article had evidence of myositis and Parvovirus B19 infection without findings of an idiopathic inflammatory myopathy [12,14,16]. Four of the five patients complained of severe bilateral calf pain [14,16], a finding commonly seen in influenza-related myositis in children [17]. Histopathologic findings of viral myositis can vary from mild inflammatory changes to severe necrosis [1]. Our case was the first to report histopathology from a biopsy in Parvovirus B19 myositis, which revealed mild acute myopathic changes showing occasional degenerating and regenerating myofibers and patchy upregulation of MHC Class I antigens. Parvovirus B19 PCR sent on muscle tissue was positive in our case and the other case that reported a biopsy [16]. Two of the five patients were treated with glucocorticoids, and the other three were treated with NSAIDs. Of the biopsy-proven cases, our patient was the only one successfully treated with NSAID monotherapy.

Parvovirus B19 has previously been proposed as a trigger of idiopathic inflammatory myopathy as well as a cause of viral myositis [18]. Interest in Parvovirus B19 as a trigger of dermatomyositis was prompted by a case reported by Chevrel et al. in 2000 of a patient with classic dermatomyositis found to have parvovirus B19 PCR positive on muscle biopsy [13]. A follow-up study including seven other patients with dermatomyositis failed to demonstrate Parvovirus B19 PCR on muscle biopsy from any individual with dermatomyositis besides the initially reported case [19], arguing against Parvovirus B19 infection as a trigger of dermatomyositis. A case-control study of 62 patients with juvenile dermatomyositis revealed that individuals with dermatomyositis had similar rates of Parvovirus B19 seropositivity as controls [20]. Subsequently, interest in Parvovirus B19 as a trigger for dermatomyositis waned. In 2012, Oliver et al. published a series of two cases of myositis associated with Parvovirus B19 infection [16] and proposed that Parvovirus B19 should still be considered as a causative factor of viral myositis. We argue that our case report and literature review provide further evidence supporting this.

There are several limitations to this paper, most notably the small sample size, which limits its applicability. Additionally, the sensitivity and specificity of Parvovirus B19 PCR sent on muscle tissue have not been established. Chevrel et al. reported negative muscle biopsy PCR tests in seven out of eight patients in their 2003 study [19], perhaps suggesting against a very high false-positive rate. Lastly, viral myositis is more common in children than in adults. Pediatric cases of Parvovirus B19 were excluded from the literature review to explore this unusual presentation in adults, so additional data from pediatric populations are not reflected. Characteristics of Parvovirus B19 infection in adults would be better evaluated in larger studies.

## Conclusions

We present a case of myositis associated with Parvovirus B19 infection and a literature review of the association between Parvovirus B19 and myositis. Several features in our case argue for myositis caused by direct invasion of muscle by Parvovirus B19, including patchy upregulation of MHC I and positive Parvovirus B19 PCR on the muscle biopsy specimen. A literature review identified four additional cases of Parvovirus B19-associated myositis and two cases of dermatomyositis. To our knowledge, our case is the second description of biopsy-proven Parvovirus B19 myositis and the first to describe specific histopathology. Further, ours is the first case of biopsy-proven Parvovirus B19 myositis to be successfully treated with NSAIDs in an adult. 
